# Prevalence, breed predispositions, and culture and sensitivity results of bacterial hepatobiliary infections in dogs in the United Kingdom

**DOI:** 10.1093/jvimsj/aalag026

**Published:** 2026-03-02

**Authors:** Frederik Allan, Aarti Kathrani, Thomas Butler, Mark Dunning, Jack Lawson, Katie E McCallum, Dan O’Neill, Lucy Yuan, Sarah Tayler

**Affiliations:** North Downs Specialist Referrals, Bletchingley RH1 4QP, United Kingdom; Department of Veterinary Clinical Sciences, Royal Veterinary College, University of London, Hertfordshire AL9 7TA, United Kingdom; Department of Veterinary Clinical Sciences, Royal Veterinary College, University of London, Hertfordshire AL9 7TA, United Kingdom; Queen’s Veterinary School Hospital, Department of Veterinary Medicine, University of Cambridge, Cambridge CB3 0ES, United Kingdom; Willows Veterinary Centre and Referral Service, Solihull B90 4NH, United Kingdom; Department of Veterinary Clinical Sciences, Royal Veterinary College, University of London, Hertfordshire AL9 7TA, United Kingdom; Queen’s Veterinary School Hospital, Department of Veterinary Medicine, University of Cambridge, Cambridge CB3 0ES, United Kingdom; Pathobiology and Population Sciences, Royal Veterinary College, University of London, Hertfordshire AL9 7TA, United Kingdom; Willows Veterinary Centre and Referral Service, Solihull B90 4NH, United Kingdom; Department of Veterinary Clinical Sciences, Royal Veterinary College, University of London, Hertfordshire AL9 7TA, United Kingdom

**Keywords:** bactibilia, bacterial hepatobiliary infection, antimicrobial resistance, cholecystitis, biliary disease

## Abstract

**Background:**

Breed predispositions for bacterial hepatobiliary infections have not been established.

**Hypothesis/Objectives:**

To determine prevalence of bacterial hepatobiliary infections in dogs presenting to referral hospitals in the United Kingdom, ascertain whether specific breed predispositions exist, and to identify associated bacterial species.

**Animals:**

One hundred twenty-six client-owned dogs diagnosed with bacterial hepatobiliary infections from 3 referral centers in the United Kingdom from a denominator cohort of 71 036 dogs.

**Methods:**

Retrospective multicenter study. Prevalence of dogs diagnosed with bacterial hepatobiliary infections was calculated. Odds ratios were calculated to establish breed predispositions. Signalment, clinicopathologic results, imaging abnormalities, bacterial culture results, gallbladder histology, treatment, and outcomes were reported.

**Results:**

Overall prevalence was 0.15% (95% CI, 0.12-0.18). Miniature Schnauzers (OR 8.95, 95% CI, 4.36-18.35, *P* < .0001) and Border terriers (OR 3.21, 95% CI, 1.06-9.70, *P* = .04) were predisposed for bacterial hepatobiliary infection compared to crossbreed dogs. *Escherichia coli* (70/156, 44.9%) and *Enterococcus* species (26/156, 16.7%) were the most frequently cultured isolates. More than 1 isolate was cultured in 33 out of 114 (28.9%) dogs. Of isolates with available susceptibility data, 55 out of 123 (44.7%) were multidrug resistant (MDR); 36 out of 123 (29.3%) were resistant to at least one fluoroquinolone, and 20 out of 123 (16.3%) were resistant to amoxicillin-clavulanate when first detected.

**Conclusions and clinical importance:**

Breed predispositions for bacterial hepatobiliary infections have been identified for Miniature Schnauzers and Border terriers. A notable proportion of bacterial isolates were MDR at first detection, but most were susceptible to amoxicillin-clavulanate.

## Introduction

Bacterial hepatobiliary infections include cholecystitis, cholangitis, and cholangiohepatitis.^1^ Although recent studies have commented that bacterial hepatobiliary infections occur more commonly in dogs than historically believed,^2–5^ to date no studies have reported prevalence. Breed predispositions for some disease processes affecting the gallbladder and biliary tract in dogs are well described, including gallbladder mucoceles (GBM) in Border terriers and Shetland Sheepdogs,^6–9^ but none are reported for bacterial hepatobiliary infections. Common etiological bacterial agents associated with bacterial hepatobiliary infections are described for cats^10^ and humans,^11^ but associated isolates and their resistance profiles in dogs are less well reported.^2,12,13^ A recent study reporting susceptibilities of bacteria cultured from bile cultures, liver tissue cultures, or a combination of both, from cats and dogs documented 40% of isolates to be multidrug resistant (MDR),^14^ but there remains a lack of literature describing resistance profiles of bacteria implicated in hepatobiliary infections in dogs.

Improved understanding of the prevalence of bacterial hepatobiliary infections in dogs, alongside identification of breeds at risk, would increase awareness and facilitate recognition of affected dogs in clinical practice. This could both improve outcomes and provide important data toward future research efforts. Awareness of bacteria and their resistance profiles would contribute to responsible (empiric) antimicrobial use by providing a stronger evidence base to inform clinical decision-making.

The current study aimed to report the prevalence of bacterial hepatobiliary infections in dogs presenting to referral centers in the United Kingdom, to determine whether breed predispositions for bacterial hepatobiliary infections exist in dogs and, if so, which specific breeds are predisposed. Secondary aims were to describe the clinical, clinicopathological, and imaging features of dogs affected by bacterial hepatobiliary infections, and to identify implicated bacterial species and their antimicrobial resistance profiles. Based on prior evidence of predisposition to other biliary diseases,^6,15,16^ it was hypothesized that Miniature Schnauzers have higher odds of bacterial hepatobiliary infections compared to crossbreed dogs, and that antimicrobial resistance would be common.

## Materials and methods

Ethical approval was granted by the Ethics and Welfare Committee of the Department of Veterinary Medicine, University of Cambridge, reference CR775. Retrospective evaluation of medical records was performed to identify all dogs with culture-confirmed or cytologically confirmed bacterial hepatobiliary infections presenting to the Queen Mother Hospital for Animals, Royal Veterinary College (Center 1) between January 1, 2010-May 31, 2023, The Queen’s Veterinary School Hospital, University of Cambridge (Center 2) between January 1, 2016-November 23, 2023, and Willows Veterinary Centre Referral Service (Center 3) between January 29, 2018-June 2, 2021. Inclusion dates were dictated by the respective digital record management systems available at each center to retrospectively search clinical data, and therefore searchable date ranges differed between institutions. Inclusion criteria required identification of bacteria from at least one of bile cytology, bile culture, gallbladder culture, liver culture, or cholelith culture, alongside available clinical records for review. Dogs were excluded if clinical records were incomplete or unavailable for review, if dogs were of non-referral status, or if bacteria were not detected.

Overall cohort data of all dogs, along with the numbers of each breed, presenting to referral services of Center 1 and Center 2 for any condition over their respective study periods were collected. To achieve this, VetCompass was used to build a denominator study group for Center 1, and cohort data were extracted from the practice management system from Center 2. It was not possible to extract overall cohort data of all dogs presenting to Center 3 and therefore dogs from this center were not included in odds ratio calculations or for reporting prevalence values. Period prevalence and 95% CI for dogs diagnosed with bacterial hepatobiliary infections in the study period were calculated for Center 1 and Center 2 as individual centers and as a combined overall group. Prevalence was compared between centers using chi-squared testing.

For the risk factor analysis, the list of breeds included as cases only covered breeds that had presented with bacterial hepatobiliary infections to at least two of three separate centers. Crossbreed dogs were used as the comparator. Breeds that presented as cases to a single center only or did not present as cases to any center were not included in the analysis as a unique breed but were grouped into a single category for analysis. Univariable odds ratios (OR) with 95% CI and *P*-values were performed using an online calculator (Medcalc Statistical Software, https://www.medcalc.org/calc/odds_ratio.php). Breeds considered significantly predisposed to bacterial hepatobiliary infections compared to crossbreed dogs were those in which the 95% confidence interval was above 1.0 and with a *P*-value of <.05.

Data extracted from clinical records at all three centers included signalment (breed, sex, neuter status, age at diagnosis), clinical signs at presentation to referral center, body condition score, clinicopathologic results at diagnosis, method of bacterial detection, bile and liver cytology results, aerobic and anaerobic bacterial culture results and susceptibilities at diagnosis (where available), modality and results of abdominal imaging studies, treatment modality including prescribed antimicrobial and hepatoprotectant medications, survival to discharge status, and follow-up data where available. Results of abdominal imaging studies were included if performed by, or under the direct supervision of, a diploma-holding radiologist. Histopathologic diagnoses were extracted if performed.

Continuous data were assessed for normality using Shapiro–Wilk and Kolmogorov–Smirnov testing. Normally distributed variables were reported using mean (±SD) and non-normal data were reported using median (interquartile range [IQR], range). Body condition scoring systems varied between and within centers, and cases were retrospectively classified as low body condition (<4/9, <3/5, or descriptive notes consistent with reduced condition), normal body condition (4-5/9, 3/5, or descriptive notes consistent with normal condition), or high body condition (>5/9, >3/5, or descriptive notes consistent with increased condition).^17,18^ Clinicopathologic reference intervals varied between and within centers over time and increases or decreases in clinicopathologic variables beyond the reference interval limits were presented as multiples of the upper or lower reference intervals, respectively. Antimicrobial resistance data were presented descriptively as proportions of isolates at the time of first positive culture that were resistant to amoxicillin/clavulanate, resistant to at least one fluoroquinolone, and isolates classified as MDR, defined as resistant to at least one antimicrobial from 3 or more different antibiotic classes.^19^

Variables with normal distribution were compared between dogs surviving to discharge and dogs that did not survive to discharge using Student’s *t*-test. Clinicopathologic variables with a non-parametric distribution were compared between dogs that survived to discharge and dogs that did not survive to discharge using Mann–Whitney U testing. Fishers-Freeman–Halton exact testing was used to compare proportions of dogs that survived to discharge and dogs that did not survive to discharge between treatment groups. *P-*value of <.05 was considered statistically significant.

## Results

### Cases

Overall, 128 dogs with bacterial hepatobiliary infections were identified (Center 1: 90, Center 2: 14, Center 3: 24) Two dogs from Center 3 were excluded from further reporting as cases, one due to incomplete clinical records, and one due to non-referral status, leaving 126 dogs for analysis.

### Prevalence

The denominator cohort for Center 1 included 54 311 dogs overall, giving a period prevalence of 0.17% (95% CI, 0.13-0.20). The denominator cohort for Center 2 included 16 725 dogs overall, giving a period prevalence of 0.08% (95% CI, 0.05-0.14). As explained in the methods, it was not possible to report prevalence for Center 3. Prevalence was significantly higher in Center 1 than Center 2 (*P* = .02).

Overall, there were 104 cases identified from 71 036 dogs seen at Centers 1 and 2, yielding a prevalence of 0.15% (95% CI, 0.12-0.18).

### Signalment and body condition score

Of 126 dogs with bacterial hepatobiliary infections, 18 (14.3%) were crossbreed dogs, 17 (13.5%) were Miniature Schnauzers, and 6 (4.8%) were Border terriers. Other affected breeds that had presented to at least 2 centers included Labrador Retrievers (5.6%, *n* = 7), Chihuahuas (4.0%, *n* = 5), Cocker Spaniels (4.0%, *n* = 5), Springer Spaniels (3.2%, *n* = 4), Cavalier King Charles Spaniels (2.4%, *n* = 3), Dachshunds (2.4%, *n* = 3), Miniature Poodles (2.4%, *n* = 3), Border Collies (1.6%, *n* = 2), Greyhounds (1.6%, *n* = 2), Jack Russell terriers (1.6%, *n* = 2), and Norfolk terriers (1.6%, *n* = 2).

Mean age at diagnosis was 8.7 years (±SD 3.1 years). Fifty-six (44.4%) dogs were female-neutered, 45 (35.7%) were male-neutered, 15 (11.9%) were male-intact, and 10 (7.9%) were female-intact. Body condition score (BCS) data at time of diagnosis at referral center were available for 101 out of 126 (80.2%) dogs. Thirteen of these dogs (12.9%) had a decreased BCS, 65 (64.4%) had a normal BCS, and 23 (22.8%) had increased BCS.

### Breed odds

A total of 71,036 dogs were presented to Centers 1 and 2 over the respective study periods. Miniature Schnauzers (OR 8.95, 95% CI, 4.36-18.35, *P* < .0001) and Border terriers (OR 3.21, 95% CI, 1.06-9.70, *P* = .04) showed increased odds of diagnosis of bacterial hepatobiliary infection compared with crossbreed dogs (Table 1). No other breeds were identified with significantly increased risk or protective effect versus crossbreeds.

**Table 1:** Number of dogs of each breed diagnosed with bacterial hepatobiliary infections and total number of dogs of each breed presenting to the Queen Mother Hospital for Animals, Royal Veterinary College (Center 1) between January 1, 2010-May 31, 2023 and The Queen’s Veterinary School Hospital, University of Cambridge (Center 2) between January 1, 2016-November 23, 2023.

**Breed**	**(Center 1)** **No. cases/no. non-cases**	**(Center 2)** **No. cases/no. non-cases**	**(Overall) No. cases/total no. non-cases**	**Odds ratio**	**95% CI**	***P***
**Crossbreed**	12/8589	3/769	15/9358	Baseline	–	–
**Border Collie**	1/1034	1/368	2/1402	0.89	0.20-3.90	.88
**Border Terrier**	**2/604**	**2/173**	**4/777**	**3.21**	**1.06-9.70**	**.04**
**Breed - other**	48/35459	2/12777	50/48236	0.65	0.36-1.15	.14
**Cavalier King Charles Spaniel**	1/1460	1/304	2/1764	0.71	0.16-3.10	.65
**Chihuahua**	3/910	1/350	4/1260	1.98	0.66-5.98	.23
**English Springer Spaniel**	3/903	1/381	4/1284	1.94	0.64-5.86	.24
**Labrador Retriever**	7/4467	1/1442	8/5909	0.84	0.36-1.99	.70
**Miniature Schnauzer**	**13/885**	**2/161**	**15/1046**	**8.95**	**4.36-18.35**	**<.0001**

Odds ratios with 95% confidence intervals for diagnosis of bacterial hepatobiliary infection in affected breeds against Crossbreed dogs are presented. Breeds with statistically significant increased risk of diagnosis of bacterial hepatobiliary infections compared to Crossbreed dogs are in bold.

### Clinical signs

Clinical signs were present in 124 out of 126 dogs (Table 2). Two cases were considered asymptomatic/subclinical, and underwent investigations due to abnormally high ALP activity (9.4 × URI and 7.8 × URI, respectively).

**Table 2:** Clinical signs reported in 126 dogs diagnosed with bacterial hepatobiliary infections, presented as number of dogs reported to display these signs alongside percentage of total cases reported to display these signs.

**Clinical sign**	**Number**	**%**
**Hyporexia**	92	73.0
**Lethargy**	90	71.4
**Vomiting**	84	66.7
**Abdominal pain**	54	42.9
**Diarrhea**	40	31.7
**Jaundice**	39	31.0
**Weight loss**	16	12.7
**Polyuria/polydipsia**	16	12.7
**Regurgitation**	4	3.2
**Concurrent immune-mediated hemolytic anemia**	4	3.2
**Weakness**	2	1.6
**Seizure**	2	1.6
**Shivering**	2	1.6
**Asymptomatic/subclinical**	2	1.6
**Weight gain**	1	0.8
**Epistaxis**	1	0.8
**Melena**	1	0.8
**Polyphagia**	1	0.8
**Hematemesis**	1	0.8
**Collapse**	1	0.8
**Dermatopathy**	1	0.8
**Flatulence**	1	0.8
**Pigmenturia**	1	0.8
**Ptyalism**	1	0.8

### Clinicopathologic abnormalities

Hematology was performed in 121 out of 126 (96%) dogs, serum biochemistry in 125 out of 126 (99.2%) dogs, CRP was measured in 67 (53.2%) dogs, and measurement of cPLI, or a cPL SNAP (IDEXX, United Kingdom), was performed in 28 (22.2%) dogs (Table 3). Of dogs in which cPLI or cPL SNAP was performed, 16 (57%) had either an abnormal cPL SNAP or cPLI >200 μg/L, of which 12 out of 16 (75%) had cPLI above 400 μg/L. DGGR lipase was increased in 8 out of 15 (53%) of dogs in which it was measured. No dogs had both DGGR lipase and cPLI measured.

**Table 3:** Clinicopathological abnormalities of dogs diagnosed with bacterial hepatobiliary infections with available clinicopathologic data for review.

**Clinicopathological abnormality (*n* = number of abnormal values/number of cases test performed in)**	**Median xRI (range xRI)**	**Below RI (*n*, (%))**	**Within RI (*n*, (%))**	**Above RI (*n*, (%))**
**WBC (*n* = 34/121)**	1.41 (1.01-4.24)	0 (0%)	87 (71.9%)	34 (28.1%)
**Neutrophils (*n* = 59/121)**	1.47 (0.24-5.54)	2 (1.7%)	62 (51.2%)	57 (47.1%)
**Evidence of left shift or toxic change (*n* = 41/121)**	n/a	2 (100%)	7 (11.3%)	32 (56.1%)
**Monocyte (*n* = 28/121)**	1.47 (0-4.83)	1 (0.8%)	93 (76.9%)	27 (22.3%)
**Lymphocytes (*n* = 29/121)**	0.69 (0.17-2.91)	26 (21.5%)	92 (76%)	3 (2.5%)
**PCV/HCT (*n* = 20/121)**	0.81 (0.27-1.25)	19 (15.7%)	101 (83.5%)	1 (0.8%)
**Albumin (*n* = 52/125)**	0.89 (0.5-0.99)	52 (41.6%)	73 (58.4%)	0 (0%)
**ALP (*n* = 118/125)**	10.47 (0.37-164.34)	2 (1.6%)	7 (5.6%)	116 (92.8%)
**ALT (*n* = 117/125)**	5.51 (0.27-136)	13 (10.4%)	8 (6.4%)	104 (83.2%)
**Bilirubin (*n* = 106/125)**	5.73 (0.25-293.75)	7 (5.6%)	19 (15.2%)	99 (79.2%)
**CRP mg/L (*n* = 63/67)**	59.9mg/L (2.7-359)	n/a	4 (6%)	63 (94%)
**Cholesterol (*n* = 97/125)**	1.38 (0.27-3.69)	25 (20%)	28 (22.4%)	72 (57.6%)
**Urea (*n* = 15/125)**	1.13 (0.74-7.37)	6 (4.8%)	110 (88%)	9 (7.2%)
**cPL >200 OR abnormal SNAP (*n* = 16/28)**	n/a	n/a	12 (42.9%)	16 (57.1%)
**DGGR lipase (*n* = 8/15)**	1.70 (0.30-24.48)	n/a	7 (46.7%)	8 (53.3%)

In the first column “n” corresponds to the number of cases in which the value was reported to be abnormal, with total number of dogs the test was performed in indicated by the value immediately following “/”. Numbers of dogs with testing results below, within or above RI are presented (*n*) alongside percentages of total cases with applicable available results (%). Abbreviations: ALP = alkaline phosphatase; ALT = alanine aminotransferase; CRP = C-reactive protein; cPL = canine pancreatic lipase immunoreactivity; DGGR = 1,2-o-dilauryl-rac-glycero-3-glutaric acid-(6'-methylresorufin) ester; HCT = hematocrit; PCV = packed cell volume; WBC = white blood cell count.

### Imaging findings and comorbidities

Abdominal imaging was performed in 120 out of 126 (95.2%) dogs (Table 4). Of these, 103 out of 120 (85.8%) dogs underwent abdominal ultrasonography and 17 out of 120 (14.2%) dogs had CT imaging. Imaging was either not performed or results were unavailable in 4 dogs, and 2 dogs had point-of-care-ultrasound only.

**Table 4:** Abnormalities identified on abdominal imaging of 120 dogs diagnosed with bacterial hepatobiliary infections.

**Imaging abnormality**	***n***	**%**
**Hepatic abnormality**	70	58.3
**Sludge/sediment**	64	53.3
**Thickened gallbladder wall**	44	36.7
**Common bile duct dilatation**	33	27.5
**Abdominal lymphadenopathy**	27	22.5
**Peritoneal effusion**	25	20.8
**Pancreatic abnormality**	25	20.8
**Irregular gallbladder wall**	20	16.7
**Cholelith**	15	12.5
**Polypoid gallbladder lesion**	11	9.2
**Thickened common bile duct**	10	8.33
**Gas in gallbladder lumen**	7	5.8
**Suspected gallbladder mucocele**	4	3.3
**Content within common bile duct**	2	1.7
**Double-layered gallbladder wall**	2	1.7
**Hypoechoic gallbladder wall**	1	0.8
**Liver mass**	1	0.8
**Stent in common bile duct**	1	0.8
**Pancreatic mass**	1	0.8
**Mineralized gallbladder wall**	1	0.8
**Pulmonary thromboembolism**	1	0.8
**Pericholedochal mass**	1	0.8

Data are presented as total number of dogs in which the abnormality was reported (*n*) alongside percentage of total dogs that underwent abdominal imaging (%). Hepatic abnormalities are grouped into a single category, as are pancreatic abnormalities.

Cholelithiasis was identified on imaging in 15 out of 120 (12.5%) cases, however, a total of 21 dogs (15.9%) had cholelithiasis, with 5 additional cases detected intraoperatively and cholelithiasis was identified on follow-up imaging in 1 dog. Other comorbidities included suspected or confirmed pancreatitis (11.9%, *n* = 15), chronic enteropathy (9.5%, *n* = 12), endocrinopathy (5.6%, *n* = 7) [diabetes mellitus (*n* = 4), hyperadrenocorticism (*n* = 1), hypothyroidism (*n* = 2)], non-hepatobiliary neoplasia (5.6%, *n* = 9) [lymphoma (*n* = 3), mast cell tumor (*n* = 1), metastatic anal sac adenocarcinoma (*n* = 1), hepatoid gland carcinoma (*n* = 1), pulmonary carcinoma (*n* = 1), unilateral adrenal mass with vascular invasion (*n* = 1), completely excised jejunal adenocarcinoma (*n* = 1)], septic peritonitis (4.8%, (*n* = 6), of which 2 had hepatic abscesses, 1 with concurrent bile peritonitis), bile peritonitis (4.8%, *n* = 6), immune-mediated disease (4%, *n* = 5) [immune-mediated hemolytic anemia (IMHA) (*n* = 4), immune-mediated polyarthritis (IMPA) (*n* = 1)], idiopathic epilepsy (2.4%, *n* = 3), hepatocellular carcinoma (0.8%, *n* = 1), sudden acquired retinal degeneration syndrome (0.8%, *n* = 1), suspected sphincter of Oddi dysfunction based on presence of plant material on bile cytology (0.8%, *n* = 1), and endocarditis (0.8%, *n* = 1).

Of dogs with non-hepatobiliary neoplasia, 3 out of 9 received chemotherapy. One dog was receiving carboplatin for incompletely excised hepatoid gland carcinoma, 1 was receiving mitoxantrone for metastatic anal sac adenocarcinoma, and 1 with pulmonary carcinoma had completed four cycles of vinorelbine within one month of detection of bacterial hepatobiliary infection. Six dogs with non-hepatobiliary neoplasia were not receiving chemotherapy, of which 5 out of 6 dogs were diagnosed with bacterial hepatobiliary infection and neoplasia concurrently, and 1 was diagnosed at restaging of a completely excised jejunal carcinoma.

Of dogs with immune-mediated disease, 3 out of 5 (60%) (IMHA [*n* = 2], IMPA [*n* = 1]) were receiving immunosuppression (prednisolone 2 mg/kg SID and azathioprine 50 mg/m^2^ EOD [*n* = 1], dexamethasone 0.4 mg/kg IV SID [*n* = 1], and prednisolone 1.5 mg/kg PO SID and chlorambucil 2 mg/kg PO EOD [*n* = 1]), and 2 out of 5 dogs (40%) (IMHA [*n* = 2]) had immune-mediated diseases suspected associative to bacterial hepatobiliary infection.

### Method of bacterial detection, bacterial culture results, and resistance profiles at first detection

Bactibilia was identified by bile culture, bile cytology, or a combination of these, in 119 out of 126 (94.4%) dogs (Table 5). In the remaining 7 dogs, bacteria were identified on gallbladder culture in 4 dogs, gallbladder and liver culture in 1 dog, liver culture in 1 dog, and cholelith and peritoneal fluid culture in 1 dog.

**Table 5:** Method of identification of bacterial hepatobiliary infection in 126 affected dogs, with specific data describing whether bile culture and cytology identified bacteria (positive) or failed to identify bacteria (negative) in dogs in which these tests were performed.

	Positive bile culture (*n* = 107)	Negative bile culture (*n* = 13)	Bile culture not performed (*n* = 6)
**Positive bile cytology (*n* = 91)**	79	12	0
**Negative bile cytology (*n* = 14)**	13	1	0
**Bile cytology not performed (*n* = 21)**	15^a^	0	6

^a^one case with positive bile culture where bile cytology was not performed had the same organism identified in concurrent positive liver and peritoneal fluid culture.

Discordant bile cytology and culture results were present in 25 out of 105 (23.8%) dogs. Antimicrobials were either being administered or had been administered within 30 days of referral in 9 out of 25 (36%) of these dogs.

A total of 156 isolates (Table 6) were cultured from 114 dogs with positive culture samples at first detection (106 dogs with positive bile cultures, 4 dogs with positive gallbladder cultures, 1 dog with positive liver and gallbladder culture, 1 dog with positive bile, liver, and peritoneal fluid culture, 1 dog with positive peritoneal fluid and cholelith culture, and 1 dog with positive liver culture).

**Table 6:** Bacterial isolates identified on positive microbiological culture samples at time of first positive culture.

**Isolate**	**Number of separate isolates cultured (*n* = 156)**	**Percentage**	**Susceptibility data available**	**Proportion resistant to amoxicillin/clavulanate**	**Proportion resistant to at least 1 fluoroquinolone**	**Proportion MDR**
***Escherichia coli***	70	44.9	62/70 (88.6%)	12/62 (19.4%)	5/62 (8.1%)	26/62 (41.9%)
***Enterococcus* spp.**	26	16.7	21/26 (80.8%)	0/21 (0%)	14/21 (66.7%)	12/21 (57.1%)
***Streptococcus* spp.**	12	7.7	12/12 (100%)	0/12 (0%)	7/12 (58.3%)	4/12 (33.3%)
***Campylobacter jejuni***	10	6.4	7/10 (70%)	1/7 (14.3%)	3/7 (42.9%)	3/7 (42.9%)
***Clostridium* spp.**	8	5.1	2/8 (25%)	0/2 (0%)	0/2 (0%)	0/2 (0%)
***Klebsiella* spp.**	7	4.5	7/7 (100%)	4/7 (57.1%)	4/7 (57.1%)	5/7 (71.4%)
***Pseudomonas aeruginosa***	3	1.9	3/3 (100%)	1/3 (33.3%)	2/3 (66.7%)	2/3 (66.7%)
***Staphylococcus pseudintermedius***	2	1.3	2/2 (100%)	0/2 (0%)	0/2 (0%)	0/2 (0%)
***Bacteroides***	2	1.3	2/2 (100%)	0/2 (0%)	1/2 (50%)	1/2 (50%)
**Unidentified gram negative rods**	2	1.3	0/2	n/a	n/a	n/a
**Unidentified gram negative bacilli**	2	1.3	1/2 (50%)	1/1 (100%)	0/1 (0%)	0/1 (0%)
**Unidentified gram positive cocci**	2	1.3	1/2 (0%)	0/1 (0%)	0/1 (0%)	1/1 (100%)
***Bacillus***	1	0.6	1/1 (100%)	0/1 (0%)	0/1 (0%)	0/1 (0%)
***Listeria monocytogenes***	1	0.6	0/1 (0%)	n/a	n/a	N/a
**Unidentified beta hemolytic anaerobe**	1	0.6	0/1 (100%)	n/a	n/a	n/a
**Unidentified coliform**	1	0.6	0/1 (0%)	n/a	n/a	n/a
**Unidentified gram positive anerobic bacilli**	1	0.6	0/1 (0%)	n/a	n/a	n/a
**Unidentified gram positive rod**	1	0.6	0/1 (0%)	n/a	n/a	n/a
**Unidentified gram positive slender rod**	1	0.6	0/1 (0%)	n/a	n/a	n/a
**Unidentified filamentous bacteria**	1	0.6	0/1 (0%)	n/a	n/a	n/a
**Unidentified lactose-fermenting coliform**	1	0.6	1/1 (100%)	1/1 (100%)	0/1 (0%)	1/1 (100%)
**Unidentified isolate**	1	0.6	0/1 (0%)	n/a	n/a	n/a

A total of 156 isolates were cultured from 114 dogs at first diagnosis. Microbiological culture results at first diagnosis are from 106 dogs with positive bile cultures, 4 dogs with positive gallbladder cultures, 1 dog with positive gallbladder and liver culture, 1 dog with positive bile, liver, and peritoneal effusion culture, 1 dog with positive cholelith and peritoneal fluid culture, and 1 dog with positive liver culture. Data are presented as individual number of isolates cultured alongside percentage of total cultured isolates.

A single isolate was cultured in 81 dogs (71.1%) and multiple isolates in 33 dogs (28.9%). The most frequently identified isolates were *Escherichia coli* (44.9%) and *Enterococcus* spp. (16.7%) (Table 6). Bacterial isolates cultured from apparently asymptomatic dogs were *Enterococcus* spp. in 1 dog, and *Clostridium* spp. in the other. Fifty-five dogs had received antibiotics within 30 days of referral.

Resistance data were available for 123 out of 156 (78.8%) isolates at first detection. Twenty isolates (16.3%) were resistant to amoxicillin/clavulanate; 36 (29.3%) were resistant to at least 1 fluoroquinolone, and 55 (44.7%) were MDR. Antimicrobials had been administered within 30 days of referral in 13 out of 18 (72.2%) dogs with isolates resistant to amoxicillin/clavulanate, 15 out of 32 (46.9%) dogs with isolates resistant to at least 1 fluoroquinolone, and 20 out of 39 (51.3%) dogs with MDR isolates.

### Gallbladder and hepatic histology

Gallbladder histology results were available for 33 out of 126 (26.2%) dogs. Findings included chronic cholecystitis (14/33, 42.4%), chronic-active cholecystitis (11/33, 33.3%), acute necrotizing or necrosuppurative cholecystitis (6/33, 18.2%), gallbladder carcinoid (1/33, 3%) and non-specific fibrosis and lymphoid follicles (1/33, 3%). Of 4 dogs with suspected mucocele on imaging, histology revealed chronic cholecystitis (*n* = 2), chronic-active cholecystitis (*n* = 1), and acute necrosuppurative cholecystitis (*n* = 1), and intraluminal contents included hemorrhage (*n* = 2), purulent material (*n* = 1), and increased mucus (*n* = 1). Hepatic histology results were available for 52/126 (41.3%) of dogs (Table 7) and hepatic cytology in 71/126 (56.3%) dogs (Supplementary Information 1).

**Table 7:** Histologic features identified in 52 dogs with available liver histology data, presented as number of dogs in which specific histologic findings were identified alongside percentage of total dogs with liver histology data with these findings.

**Histologic diagnosis**	**Number**	**Percentage**
**Cholangiohepatitis** ^**a**^	14	26.9
**Chronic hepatitis**	9	17.3
**Non-specific reactive hepatopathy**	8	15.4
**Other** ^**b**^	5	9.6
**Vacuolar hepatopathy**	4	7.7
**Cholangitis** ^**c**^	2	3.8
**Hepatic abscess** ^**d**^	2	3.8
**Vascular disease** ^**e**^	2	3.8
**Abscessating hepatitis**	1	1.9
**Cirrhosis**	1	1.9
**Copper-associated hepatopathy**	1	1.9
**Fibrinosuppurative hepatitis**	1	1.9
**Neoplasia (hepatocellular carcinoma)**	1	1.9
**Nodular hyperplasia**	1	1.9

Abbreviations: LP = lymphoplasmacytic; N = neutrophilic.

^a^Cholangiohepatitis—14 (LP and neutrophilic 4; LP/N/histiocytic—2; neutrophilic—4; neutrophilic and concurrent parasitic granuloma—1; “chronic active”—1; “mixed”—1; “subacute”—1).

^b^Other—5 (diffuse hepatic atrophy and biliary hyperplasia—1; biliary hyperplasia and arterial duplication—1; multifocal hepatopathy with single cell necrosis, portal fibrosis, oval cell hyperplasia, centrilobular fibrosis—1; portal biliary and arterial hyperplasia with periacinar hepatocellular degeneration, disseminated lipogranulomas—1; massive necrosis and marked biliary hyperplasia—1).

^c^Cholangitis—2 (neutrophilic chronic-active, 1; neutrophilic/LP chronic active, 1).

^d^Hepatic abscess—2 (concurrent mixed cholangitis and vacuolar hepatopathy—1; concurrent non-specific reactive hepatopathy—1).

^e^Vascular—2 (portal vein hypoplasia—1; microvascular hepatopathy—1).

### Treatment and outcomes

#### Medical

Overall, 88 out of 126 (69.8%) dogs were managed conservatively. Antimicrobials were administered to 83 out of 88 (94.3%) dogs (Table 8). Duration of antimicrobial therapy was available for 60 out of 83 dogs, with a median of 28 days (range 1-61 days). Five dogs did not receive antimicrobials (Supplementary information 2).

**Table 8:** Antimicrobial treatments and their duration (where data available) administered to dogs with bacterial hepatobiliary infections.

**Antimicrobial**	**Medically managed dogs (*n* = 88)**	**Median duration of initial treatment/median duration of treatment with alternative antimicrobial; days (range)**	**Surgically managed dogs (*n* = 28)**	**Median duration of treatment/median duration of treatment with alternative antimicrobial; days (range)**	**Dogs with medical management followed by surgery (*n* = 10)**	**Median duration of treatment/median duration of treatment with alternative antimicrobial; days (range)**
**Single agent only**	59	28 (3-51)	19	24.5 (7-70)	3	42 (28-68)
**Amoxicillin/clavulanate**	45	28 (3-51)	15	21 (7-70)	3	42 (28-68)
** Marbofloxacin**	8	28 (4-28)	1	n/a	0	n/a
** Enrofloxacin**	3	34.5 (24-45)	2	n/a	0	n/a
** Metronidazole**	2	21	0	n/a	0	n/a
** TMPS**	1	n/a	0	n/a	0	n/a
** Doxycycline**	0	n/a	1	28	0	n/a
**Single agent with change in antimicrobial based on culture result**	5	28 (8-49)/28 (28-42)	0	n/a	2	25, n/a/42, n/a
** Amoxicillin/clavulanate to enrofloxacin**	2	8, 49/28, n/a	0	n/a	0	n/a
** Cephalexin to amoxicillin/ clavulanate**	1	14/28	0	n/a	0	n/a
** TMPS to amoxicillin/clavulanate**	1	5/28	0	n/a	0	n/a
** Amoxicillin/clavulanate to marbofloxacin**	1	28/42	0	n/a	1	25/42
** Amoxicillin/clavulanate to TMPS**	0	n/a	0	n/a	1	n/a
**Single agent with change in antimicrobial due to lack of clinical response**	2	21 / 14	0	n/a	1	6/n/a
** Amoxicillin/clavulanate to marbofloxacin**	2	21, n/a/14, n/a	0	n/a	0	n/a
** Cephalexin to marbofloxacin**	0	n/a	0	n/a	1	6/n/a
**Combination therapy**	13	32.5 (5-61)	9	9 (2-31)	4	42, 56
** Amoxicillin/clavulanate and marbofloxacin**	2	28 (both)	2	28, 31	2	n/a
** Amoxicillin/clavulanate and enrofloxacin**	2	35, 59	4	7 (7-19)	0	n/a
** Amoxicillin/clavulanate and metronidazole**	1	14	0	n/a	0	n/a
** Amoxicillin/clavulanate and doxycycline**	1	39	0	n/a	0	n/a
** Amoxicillin/clavulanate and cefuroxime**	0	n/a	1	9	0	n/a
** Enrofloxacin and metronidazole**	2	30, 35	1	n/a	0	n/a
** Marbofloxacin and metronidazole**	1	5	0	n/a	1	42
** Marbofloxacin and doxycycline**	1	42	0	n/a	0	n/a
** Marbofloxacin and clindamycin**	1	61	0	n/a	1	56
** Enrofloxacin and cephalexin**	1	n/a	0	n/a	0	n/a
** Enrofloxacin and cefuroxime**	1	12	0	n/a	0	n/a
** Amoxicillin/clavulanate, enrofloxacin and metronidazole**	0	n/a	1	2	0	n/a
**Combination therapy with a change in treatment based on culture result**	2	5, 28/28, 28	0	n/a	0	n/a
** Marbofloxacin and metronidazole to amoxicillin/ clavulanate**	1	5/28	0	n/a	0	n/a
** Marbofloxacin to amoxicillin/ clavulanate and metronidazole**	1	28/28	0	n/a	0	n/a
**Combination therapy with a change in treatment due to lack of clinical response or reason not stated**	2	1, 28/28	0	n/a	0	n/a
** Amoxicillin/clavulanate and enrofloxacin to amoxicillin/clavulanate and marbofloxacin**	1	28/28	0	n/a	0	n/a
** Amoxicillin/clavulanate to marbofloxacin and clindamycin**	1	1/n/a	0	n/a	0	n/a
**No antimicrobials received**	5	n/a	0	n/a	0	n/a

Hepatoprotectant medications included ursodeoxycholic acid (UDCA) in 60 dogs and SAMe/silybin in 42 dogs (38 had both concurrently).

A total of 73 out of 83 (88%) of antimicrobial-treated dogs survived to discharge. Follow-up data is available for 69 of these dogs (median 141 days, range 7-1755), of which 78% (54/69) were reported to have resolution of clinical signs without relapse, 6% (4/69) had at least one relapse of clinical signs that was successfully managed, 3% (2/69) had a relapse that resulted in euthanasia, and 13% (9/69) had clinical signs attributable to ongoing disease at follow-up. Median survival in dogs that survived to discharge and subsequently died or were euthanized is 176 days (range 7-1755 days), and median time to last follow up in surviving dogs is 110 days (range 16–1326 days). Median time from diagnosis to death in dogs not surviving to discharge was 4.5 days (range 3-14 days). Causes of death were euthanasia due to clinical status or deterioration (*n* = 6), due to septic peritonitis (*n* = 2), due to prognosis associated with concurrent cutaneous epitheliotrophic lymphoma (*n* = 1), and death due to suspected bilirubin encephalopathy (*n* = 1).

Of the five dogs that did not receive antimicrobials, 2 did not survive to discharge (concurrent IMHA, 1 septic shock), and the other 3 survived to discharge and did not experience recurrence of clinical signs (one died after 141 days, and 2 were alive at last follow up of 64 and 118 days).

Eighteen medically managed dogs had follow-up bile sampling performed. This documented bactibilia in 14 out of 18 (78%) dogs, of which 10 (71%) dogs were considered subclinical and 4 (29%) symptomatic. Of dogs with positive follow-up bile sampling, 13 out of 14 had positive bile cultures and 1 out of 14 had positive bile cytology with negative culture. A single follow-up culture was performed in 12 out of 18 dogs and two follow-up cultures were performed in 6 out of 18 dogs (Supplementary Information 1). Median time of first follow-up culture was 44 days (range 17-117) after initial culture, and median time of second follow-up culture was 216 days (range 57-238) after initial culture.

Of dogs with positive follow-up bile cultures, 8 out of 13 were receiving antimicrobials at time of sampling. Of 4 dogs with negative follow-up sampling, 3 out of 4 were receiving antimicrobials at time of sampling and 1 dog had received antimicrobials within 24 h of sampling. The 1 dog with discordant cytology and culture results was not receiving antimicrobials at time of repeat sampling.

Of medically managed dogs with data confirming alive or deceased status at least seven days post-diagnosis, 76 out of 84 (90%) were alive at 7 days, 35/52 (67%) dogs were alive at 6 months, and 23 out of 46 (50%) alive at 1 year. Causes of death in dogs surviving to discharge were available for 16 dogs. Causes of death were related to a hepatobiliary cause in 3 dogs, and unrelated in 13 dogs. Causes unlikely related to biliary infection included neurological disease or signs (*n* = 5), neoplasia (*n* = 4), and one each of septic peritonitis, acute onset hypoglycemic collapse, euthanasia due to sudden onset lameness with weight loss, and euthanasia due to a “catastrophic incident.”

#### Surgical

Twenty-eight dogs (22.2%) were managed surgically. Twenty-five dogs (89%) had a single procedure, 2 (7%) dogs had two procedures, and 1 (4%) dog had three procedures.

In dogs that underwent a single surgical procedure, procedures performed included cholecystectomy (*n* = 10, 40%), cholecystectomy and duodenotomy (*n* = 8, 32%), duodenotomy and choledochal stent placement (*n* = 4, 16%), cholecystectomy and stent placement (*n* = 1, 4%), liver lobectomy (*n* = 1, 4%), and ultrasound-assisted stent placement (*n* = 1, 4%).

Reasons for initial surgical procedure included suspected biliary obstruction (*n* = 6, 21%), bile peritonitis (*n* = 5, 18%), septic peritonitis (*n* = 3, 11%), obstructive cholelithiasis (*n* = 3, 11%), suspected mucocele (symptomatic) (*n* = 3, 11%), concern for GB rupture (*n* = 2, 7%), GB mass lesion (*n* = 1, 4%), suspected mucocele and obstruction (*n* = 1, 4%), concern for high risk of peritonitis post-sampling (*n* = 1, 4%), and reason was not specified in 3 (11%) dogs. Details regarding dogs undergoing multiple surgical procedures is available in Supplementary Information 2.

All surgically managed dogs also received antimicrobial treatment (Table 8). Duration of antimicrobial therapy was available for 15 dogs, with a median of 19 days (range 2-70 days). Specific hepatobiliary medications included UDCA in 13 dogs and SAMe/silybin in 9 dogs (all 9 receiving UDCA concurrently).

Seven dogs (25%) that underwent surgery did not survive to discharge. Causes of death included euthanasia due to development of septic peritonitis (*n* = 2), due to clinical deterioration post-operatively (*n* = 1), due to multiple organ dysfunction syndrome (*n* = 1), and due to a combination of congestive heart failure, melting corneal ulcers, aspiration pneumonia and pancreatitis (*n* = 1), and death due to systemic inflammatory response syndrome (*n* = 1), and due to septic peritonitis (*n* = 1). Median survival of dogs not surviving to discharge was 9 days (range 2-19 days).

Twenty-one dogs (75%) survived to discharge. Follow-up data was available for 16 dogs (median 78 days, range 7-2334). Ten dogs had complete resolution of clinical signs with no relapse episodes, 2 had recurrent episodes that were successfully managed with repeat surgery, 1 dog had a relapse of signs and was euthanized, 3 dogs had ongoing clinical signs, and 5 dogs were lost to follow-up after discharge.

Four dogs had follow-up hepatobiliary sampling, with a single follow-up culture performed in 3 out of 4 dogs and two follow-up cultures in 1 dog (Supplementary Information 1). Positive cultures occurred in all 4 dogs, with bile culture in 2 out of 4 dogs, gallbladder tissue culture in 1 dog, and liver aspirate culture in 1 dog. Three dogs were receiving antimicrobials at the time of follow-up sampling. Time between initial culture and first follow-up culture was available for two dogs, performed at 30 days in 1 dog and 346 days in the other. Time between initial culture and second follow-up culture in 1 dog was 94 days.

Of surgically managed dogs, 21 out of 23 (91%) were alive at 7 days, 6 out of 15 (40%) were alive at 6 months, 4 out of 14 (29%) were alive at 1 year (Table 9).

**Table 9:** Proportions of dogs with bacterial hepatobiliary infections alive at 7 days, 6 months, and 1 year after diagnosis.

	**7 days**	**6 month**	**1 year**
**Medical (*n* = 88)**	76/84 (90.5%)	35/52 (67.3%)	23/46 (50%)
**Surgical (*n* = 28)**	21/23 (91.3%)	6/15 (40%)	4/14 (28.6%)
**Delayed surgical (*n* = 10)**	7/7 (100%)	3/4 (75%)	1/3 (33.3%)
**Overall (*n* = 126)**	104/114 (91.2%)	44/71 (62.0%)	28/63 (44.4%)

Data are presented as dogs alive at the indicated time period/total dogs confirmed deceased plus dogs confirmed alive with follow-up available at the indicated time period. Data are presented for individual treatment groups and for total dogs at each time period.

#### Medical management followed by surgery

Ten dogs (7.9%) were initially managed conservatively before progressing to require surgical intervention. All dogs survived to discharge.

Duration of medical management before surgery was available in 9 out of 10 (90%) dogs, with a median of 68 days (range 20-598 days). All dogs received antimicrobial treatment (Table 8), 7 (70%) received UDCA, and 6 (60%) received SAMe/silybin (5/6 alongside UDCA). Duration of initial antimicrobial therapy was available for 7 dogs, with a median of 42 days (range 6-68 days).

Reasons for surgical intervention were recurrent episodes of cholecystitis (*n* = 3, 30%), relapse of signs with suspected biliary obstruction (*n* = 3, 30%), persistent/ongoing bactibilia (*n* = 2, 20%), persistent ultrasonographic gallbladder abnormalities (*n* = 1, 10%), and relapse of signs and a choledocholith (*n* = 1, 10%). Repeat sampling had been performed in 9 (90%) dogs before or at the time of surgery, with positive cultures in 6 (66.7%) dogs (Supplementary Information). One dog’s first positive culture was *Clostridium perfringens* isolated from gallbladder tissue culture taken at time of surgery, having had a negative bile culture at start of treatment while receiving antimicrobials.

Five dogs had multiple repeat cultures performed before surgery. Of these, 3 out of 5 had two repeat cultures and 2 out of 5 had three repeat cultures. In the 3 dogs with two cultures performed before surgery, cultures were positive on both occasions in 2 out of 3 dogs, and negative on both occasions in 1 dog. In dogs with 3 follow-up cultures before surgery, culture was positive on 2 out of 3 occasions in 1 dog, and 3 out of 3 occasions in the other. No dogs had follow-up culture performed after surgery.

All dogs survived to discharge post-operatively. Seven (70%) had complete resolution of clinical signs (median 51.5 days, range 20-1023) and 3 (30%) were lost to follow-up. Three (30%) dogs have since died (median 362 days, range 27-1023 days), median time to last follow-up in surviving dogs was 34 days (range 20-69 days) in 3 dogs and number of days to follow-up was not available in 1 dog. Causes of death were unrelated to biliary infection in 2 dogs (neoplasia in 1, acute kidney injury in 1) and portal hypertension in 1 dog. Of medically managed dogs progressing to surgery with follow-up available, 7 out of 7 dogs were alive at 7 days, 3 out of 4 dogs alive at 6 months, and 1 out of 3 dogs alive at 1 year.

#### Factors associated with survival to discharge

**Figure 1:**
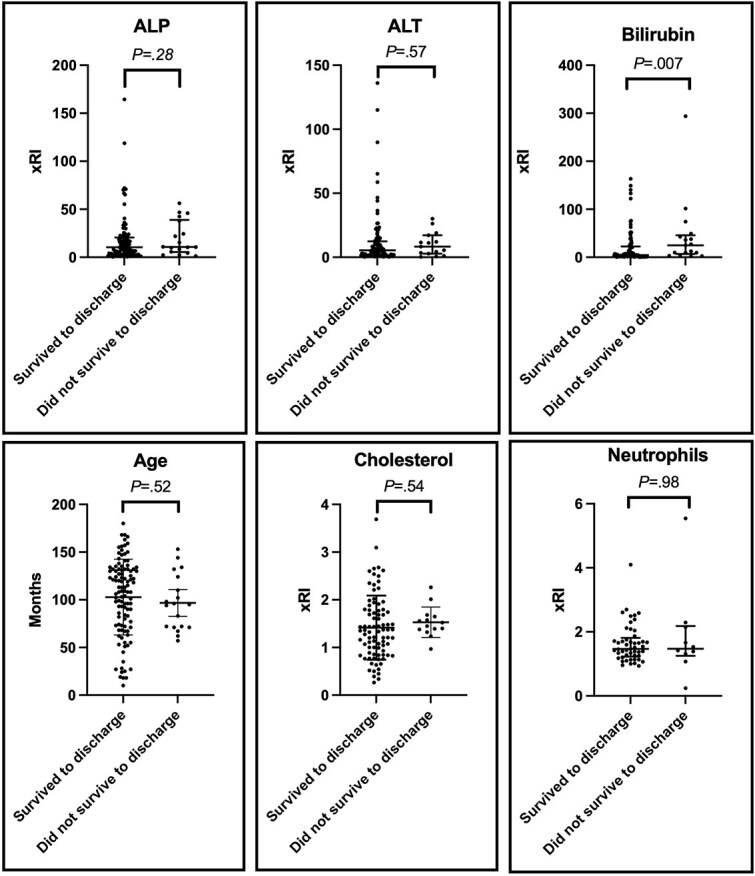
Serum ALP activity, ALT activity, and bilirubin and cholesterol concentrations, and age in months and neutrophil count of dogs diagnosed with bacterial hepatobiliary infections compared between dogs surviving to discharge against dogs that did not survive to discharge. As reference intervals varied between and within centers, results for biochemical variables and neutrophil count are presented as a proportion of the upper or lower limit of the reference interval (xRI). Elbow brackets illustrate P values of Mann–Whitney U tests for ALP activity, ALT activity, bilirubin concentration and neutrophil count between dogs surviving to discharge and dogs that did not survive to discharge; and of Student’s *t*-test for age (in months) and cholesterol concentrations between dogs surviving to discharge and dogs that did not survive to discharge. Error bars indicate range for ALP activity, ALT activity, bilirubin concentration and neutrophil counts, and interquartile range for age and cholesterol concentrations. Abbreviations: ALP = alkaline phosphatase; ALT = alanine aminotransferase; xRI = proportion of reference interval limit.

Bilirubin was significantly higher in dogs that did not survive to discharge than those that survived to discharge (median 24.71xRI [range 1.95-293.8] vs 4.43xRI [0.25-163.4], (*P* = .007)). There were no differences in age (*P =* .52), ALP activity (*P* = .28), ALT activity (*P* = .57), cholesterol (*P* = .54), or neutrophils (*P* = .98), between dogs that did not survive to discharge and did survive to discharge (Figure 1). There were no differences in proportions of dogs surviving to discharge between treatment groups (*P* = .17).

## Discussion

This study has identified Miniature Schnauzers and Border terriers as having a greater risk for bacterial hepatobiliary infection compared to crossbreed dogs, reports prevalence of bacterial hepatobiliary infections in dogs, and describes implicated bacterial isolates and their resistance profiles at first detection.

*E. coli* and *Enterococcus* species being the most frequently identified isolates aligns with findings of previous studies in dogs,^2,3,5,12,13^ and is similar to reports of cats and humans with cholangitis.^10,11,20^ This adds further evidence to proposals that the etiology of bacterial hepatobiliary infections in most dogs is likely either due to ascending biliary infections or translocation of enteric bacteria.^5^ Some bacterial species are challenging to culture,^21^ and that traditional culture methods could underestimate or exclude bacterial species that other methods of bacterial detection would identify. These factors could account for the dogs with positive cytologic identification of bactibilia alongside negative culture in the present study. Limitations with traditional culture methods could also overestimate the prevalence of readily cultured bacterial species, which might have led to skewed identification of bacterial populations in the present study.

Antimicrobial resistance was common, with almost 45% of isolates being MDR at first detection, similar to a recent study where MDR isolates were present in 44% of dogs in which bile or liver tissue samples were cultured.^14^ These findings emphasize the importance of bacterial culture and susceptibility testing to inform antimicrobial use. Because of the retrospective nature of this study, with variability in susceptibility profiles performed, description of antimicrobial resistance data was simplified to presence or absence of resistance to amoxicillin/clavulanate, resistance to one or more fluoroquinolones, and resistance to one antimicrobial from three or more different antimicrobial classes (MDR).^19^ It is important to be aware that some bacterial species have intrinsic resistances to specific antimicrobials which impacts assessment of MDR status. Despite a large proportion of isolates being classified as MDR, only 16.3% were resistant to amoxicillin/clavulanate. This supports the British Small Animal Veterinary Association PROTECTME guidelines^22^ recommendation for use of amoxicillin/clavulanate while pending culture and susceptibility results.

Factors proposed to contribute to development of bacterial hepatobiliary infections include cholestasis, underlying gallbladder disease, immunosuppression, and extrahepatic biliary duct obstruction,^23,24^ but risk associated with breed has not been reported. Breed predispositions exist for several hepatobiliary disease processes, with Miniature Schnauzers and Border terriers both having an increased risk of developing GBM.^6,9^ While it is interesting to note breed predispositions might exist for bacterial hepatobiliary infections, further studies are required to investigate potential reasons for this. Multiple comorbidities were identified in this cohort that could have contributed to development of hepatobiliary infection. A notable proportion of dogs had cholelithiasis, which could have contributed to development of infection through biliary obstruction or gallbladder wall injury, or could have formed secondary to cholestasis or chronic infection.^25^ A number of dogs were either receiving immunosuppressive medications, had received chemotherapy, or had concurrent endocrinopathies. Endocrinopathies are a risk factor for bacterial hepatobiliary infections in humans,^26^ and for hepatobiliary diseases in dogs including GBM^8^ and vacuolar hepatopathy.^27^ It is possible immunosuppression secondary to these factors could have contributed to infection. Finally, enteric inflammation has been suggested to play a role in development of hepatobiliary infections,^23^ and concurrent chronic enteropathies were reported in nearly 10% of dogs in this study. Further research is required to investigate the potential role of these factors in development of hepatobiliary infections.

Chronic cholecystitis was the most frequently identified histologic feature in cholecystectomised dogs in the current study. Chronic cholecystitis is documented in dogs with cholelithiasis,^28^ GBM,^29,30^ hemocholecysts,^31^ and gallbladder sludge.^29^ Bacterial antigenic stimulation is postulated to play a role in development of chronic cholecystitis with histochemical detection of bacteria in 29% of cholecystectomised dogs in one study,^29^ and could be supported by the frequent finding of chronic cholecystitis in dogs in our study. Alternatively, chronic cholecystitis might predispose to development of secondary hepatobiliary bacterial infections. Chronic cholecystitis might be underrecognized in dogs with bacterial hepatobiliary infections, given that not all cases undergo cholecystectomy, and it is important to consider that medical management was the sole treatment strategy in most cases in our study which might underestimate frequency of histologic features.

While cholangiohepatitis was the most commonly identified feature in dogs in the present study, this was identified in less than a third of cases. Approximately half of dogs with cholangiohepatitis in this study had either a chronic, acute-on-chronic, or chronic-active process. This suggests that hepatobiliary infections might represent secondary opportunistic infections in some dogs. Similarly, chronic hepatitis was documented in some dogs, alongside individual cases of copper-associated hepatopathy and cirrhotic liver disease. Bacterial infection is a recognized, although rare, complication of chronic hepatitis in dogs^32^ and chronic liver disease might have contributed to development of hepatobiliary infection in these dogs.

Although the current study describes a large number of dogs with bacterial hepatobiliary infections to date and reports breed predispositions, there are limitations. First, the retrospective nature of this study is associated with inherent limitations resulting from non-standardized case management, including variation in performed investigations, treatment modalities, case management, and follow-up. Inconsistent treatment strategies and follow-up data meant that assessment of whether any specific treatment modality impacted long-term outcome or survival time was not possible. Due to the retrospective multicenter nature of this study, data describing total numbers of dogs where bile cytology or culture of bile or relevant tissues had been performed and were negative and unavailable. Therefore, the overall proportion of positive cytology or culture results was not calculable. Following on from this, measures of diagnostic accuracy of bile cytology and culture of relevant tissues were also not available, which could have led to incorrect exclusion of affected dogs. Another limitation is that the assessed date periods differed between centers. Given antimicrobial resistance profiles change over time, it is relevant to note that fluctuation of antimicrobial resistance within and between centers could have impacted our results. Miniature Schnauzers and Border terriers are breeds with well reported predispositions to disease processes affecting the biliary tree including pancreatitis,^16^ hyperlipidemia,^15^ and GBM.^6^ This might have influenced clinical decision-making and resulted in increased numbers of dogs of these breeds undergoing sampling of relevant tissues compared to dogs of other breeds. To counter potential bias, multiple centers were recruited, and odds ratios were calculated against a large, robust denominator cohort from two separate referral centers. However, one of the centers did not have a denominator cohort available, and so could not be included in the prevalence and risk factor analysis. It is possible therefore that other predispositions exist which might become clear in future studies. Prevalence of bacterial hepatobiliary infections differed statistically significantly between institutions. This might reflect institutional bias toward different caseloads depending on different specialisms and focus. Multiple centers were enrolled to account for such biases compared to single center analysis with the aim of achieving a more generalizable overall prevalence value. However, residual bias could still exist, especially given the lack of a denominator cohort from one of the three centers in the study. A statistical limitation of this study is that mixed-effects models or generalized estimating equations using hospital as a clustering variable were not performed. Therefore, it is possible that this could have inflated type I error rates and underestimated standard errors in our study, which would be of importance if systematic differences between centers in case management, patient populations, or diagnostic practices were present. Finally, data in the current study were from referral centers and so referral bias might affect generalizability of these referral results to the wider primary care practice population.^33^

In conclusion, our findings suggest breed predispositions for bacterial hepatobiliary infections in Miniature Schnauzers and Border terriers. *E. coli* and *Enterococcus* species were the most common isolates identified, and while a significant proportion of bacteria were MDR, the majority were susceptible to amoxicillin/clavulanate when first cultured.

## Supplementary Material

aalag026_Supplemental_Files

## Data Availability

The data underlying this article will be shared on reasonable request to the corresponding author.

## Off-label antimicrobial declaration

Authors declare no off-label use of antimicrobials.

## Institutional animal care and use committee or other approval declaration

Approved by the University of Cambridge Department of Veterinary Medicine Ethics review panel, reference CR775. As retrospective data specific ethical approval was not required from Royal Veterinary College ethics review board.

## Abbreviations

GBM: gallbladder mucoceles
MDR: multidrug resistant
UK: United Kingdom
OR: odds ratios
IQR: interquartile range
BCS: body condition score
IMHA: immune-mediated hemolytic anemia
IMPA: immune-mediated polyarthritis
UDCA: ursodeoxycholic acid
