# Supplementary material for: Prevalence, breed predispositions, and culture and sensitivity results of bacterial hepatobiliary infections in dogs in the United Kingdom
Source: J Vet Intern Med. 2026 Mar 2;40(2):aalag026. doi: 10.1093/jvimsj/aalag026 (PMC12952289; doi:10.1093/jvimsj/aalag026)
Supplement: aalag026_Supplemental_Files [file aalag026_supplemental_files.zip › Supplemental_info_2_post_review_16.8_aalag026.docx]

Supplementary information 2

**Treatment groups**

**Medically managed dogs that did not receive antimicrobials (n=5)**

Of the 3 dogs that survived to discharge, one was treated with IVFT, UDCA, paracetamol, maropitant and low-fat diet and had an improvement in clinical signs and mild improvement in biochemical parameters at 3 weeks; and 2 had resolution of clinical signs at the time of discharge with supportive treatment only. Treatment in these two cases included IVFT, anti-nausea medications (ondansetron in 1, maropitant in the other), omeprazole, sucralfate and fenbendazole in 1, and placement of an oesophagostomy tube with assisted enteral feeding in 1. Of the 2 dogs that did not survive to discharge, one was treated with prednisolone, clopidogrel, and a packed red blood cell transfusion for suspected IMHA and died of complications relating to IMHA, and the other was treated with UDCA and SAMe/silybin and experienced cardiopulmonary arrest due to septic shock shortly after investigations were performed. Bacteria cultured in dogs not treated with antimicrobials were *Campylobacter* (n=2), *Enterococcus spp.* (n=1), *Klebsiella pneumoniae* (n=1) and *Streptococcus* spp. (n=1).

**Surgical**

In dogs undergoing two surgical procedures, one underwent hepatic abscess debridement followed by cholecystectomy and duodenotomy after 22 days, and one dog underwent a liver lobectomy for a hepatic abscess followed by a cholecystectomy for septic bile peritonitis with cholelithiasis after 347 days.

One dog initially had a stent placed for EHBDO (initially negative for bacterial hepatobiliary infection) and after 132 days developed common bile duct obstruction with cholelithiasis (positive bile culture at this time) and underwent cholecystoduodenostomy, duodenotomy and choledochotomy, followed by revision surgery after another 4 days due to septic peritonitis.

Of dogs that survived to discharge, 3 have died since (median 78d, range 7-287days), and 5 were lost to follow up. Of dogs for which follow up is available, median time of follow-up is 95.5 days (range 9-2334 days). Time of follow-up was not available for one dog which was reported to be doing well approximately 18 months after surgery.
