# Supplementary material for: Prevalence, breed predispositions, and culture and sensitivity results of bacterial hepatobiliary infections in dogs in the United Kingdom
Source: J Vet Intern Med. 2026 Mar 2;40(2):aalag026. doi: 10.1093/jvimsj/aalag026 (PMC12952289; doi:10.1093/jvimsj/aalag026)
Supplement: aalag026_Supplemental_Files [file aalag026_supplemental_files.zip › Supplementary_info_1_post_review_16.8_aalag026.docx]

**Supplementary Information 1**

Supplementary information Table 1: Cytologic abnormalities identified on cases with available hepatic cytology results.

| **Cytologic finding** | **Number** | **Percentage** |
| --- | --- | --- |
| **Evidence of cholestasis** | 49 | 69.0% |
| **Vacuolar change** | 40 | 56.3% |
| **Neutrophilic inflammation** | 25 | 35.2% |
| **Mixed/other inflammatory population** | 20 | 28.2% |
| **Extramedullary hematopoeisis** | 9 | 12.7% |
| **Necrosis** | 7 | 9.9% |
| **Hepatocyte atypia/dysplasia** | 7 | 9.9% |
| **Biliary hyperplasia** | 5 | 7.0% |
| **Bacterial sepsis** | 3 | 4.2% |
| **Neoplasia** | 2 | 2.8% |
| **Erythrophagia** | 1 | 1.4% |
| **Unremarkable cytology** | 1 | 1.4% |

Supplementary information Table 2: Bacterial isolates and their antimicrobial resistance patterns identified on positive follow-up microbiologic cultures after initial treatment.

| **Bacterial isolates** | **Persistent/repeat infection with same organism** | **Reinfection with a new organism** | | **Number of isolates with susceptibility data available** | **Proportion resistant to amoxicillin/clavulanate** | **Proportion resistant to at least 1 fluoroquinolone** | **Proportion MDR** | **Proportion with progressive antimicrobial resistance compared to first identification** | |
| --- | --- | --- | --- | --- | --- | --- | --- | --- | --- |
| **Medically managed dogs (n=13/18)** | | | | | | | | | |
|  | **Isolates identified in 9 dogs with one positive follow-up culture** | | | | | | | | |
| *Escherichia coli (n=6)* | 3 | 3 | | 3/6*(50%)  1/3 partial | 2/3 (66.7%) | 2/2 (100%) | 2/2 (100%) | n/a (new organisms) | |
| *Enterococcus spp. (n=2)* | 0 | 2 | | 2/2 (100%) | 1/2 (50%) | 2/2 (100%) | 1/2 (50%) | n/a (new organisms) | |
| *Morganella morganii (n=1)* | 0 | 1 | | Partial | n/a | 1/1 (100%) | n/a | n/a (new organism) | |
| *Clostridium perfringens (n=1)* | 0 | 1 | | 0 | n/a | n/a | n/a | n/a | |
|  | **Isolates identified in 4 dogs with two positive follow-up cultures** | | | | | | | | |
| *Escherichia coli (n=6)* | 1 | 5 | | 5/6 (83.3%)*  *2/5 partial | 3/5 (66.7%) | 5/5 (100%) | 3/3 (100%) | 4/5 (80%) | |
| *Enterococcus spp. (n=2)* | 1 | 1 | | 1/2 (50%) | 0/1 (0%) | 0/1 (0%) | 0/1 (0%) | n/a | |
| *Klebsiella spp. (n=1)* | 1 | 0 | | 1 (100%) | 1/1 (100%) | 1/1 (100%) | 1/1 (100%) | 1/1 (100%) | |
| *Pseudomonas spp. (n=1)* | 0 | 1 | | 0 | n/a | n/a | n/a | n/a | |
| *Unidentified coliform (n=1)* | 0 | 1 | | 1/1 (100%) | 1/1 (100%) | 1/1 (100%) | 1/1 (100%) | 1/1 (100%) | |
| **Dogs that received medical management before progressing to surgery (n=6/9)** | | | | | | | | | |
|  | **Isolates identified in 2 dogs with one positive follow-up culture** | | | | | | | | |
| *Clostridium perfringens (n=1); first detection of bacteria in this dog* | unknown | unknown | | n/a | n/a | n/a | n/a | n/a | |
| *Streptococcus spp. (n=1)* | No | Yes | | 1 | n/a | n/a | n/a | n/a | |
|  | **Isolates identified in 3 dogs with two positive follow-up cultures** | | | | | | | | |
| *Escherichia coli (n=6)* | Initial culture negative in 1 dog | 5 | | 6/6 (100%) | 6/6 (100%) | 4/6 (66.7%) | 6/6 (100%) | 4/5 (80%) | |
| *Enterococcus spp. (n=1)* | 0 | 1 | | 1 (100%) | 0/1 (0%) | 0/1 (0%) | 1/1 (100%) | n/a | |
| *Proteus spp. (n=1)* | 0 | 1 | | 1 (100%) | 1/1 (100%) | 1/1 (100%) | 1/1 (100%) | n/a | |
|  | **Isolates identified in 1 dog with three positive follow-up cultures** | | | | | | | | |
| *Escherichia coli (n=3)* | Initial culture at diagnosis revealed multiple unidentified bacterial species | 2 | 3/3 (100%)*  *partial resistance data in 1/3 | | 3/3 (100%) | 3/3 (100%) | 2/2* (100%)*partial resistance data in 1/3 | | n/a |
| *Enterococcus spp (n=5)* |  | 3 | 5/5* (100%)  *partial resistance data in 2/5 | | 2/5 (40%) | 4/5 (80%) | 3/3* (100%)*partial resistance data in 2/5 | | 1/3 (33%) |
| *Klebsiella spp. (n=1)* |  | n/a | 1/1 (100%) partial resistance data | | 1/1 | 1/1 | n/a | | n/a |
| *Unidentified coliforms (n=1)* |  | n/a | 1/1 (100%) | | 1/1 (100%) | 1/1 (100%) | 1/1 (100%) | | n/a |
| **Surgically managed dogs (n=4/4)** | | | | | | | | | |
|  | **Isolates identified in 3 dogs with one positive follow-up culture** | | | | | | | | |
| *Escherichia coli (n=1)* | 1 | 0 | | 1 | 1 (100%) | 1 (100%) | 1 (100%) | 1 (100%) | |
| *Enterococcus spp. (n=2)* | 0 | 2 | | 1/2 (50%) | 0/1 (0%) | 0/1 (0%) | 0/1 (100%) | n/a | |
| *Lactose-fermenting coliform (n=1)* | 0 | 1 | | Partial | 0/1 (0%) | 1/1 (100%) | n/a | n/a | |
|  | **Isolates identified in 1 dog with two positive follow-up cultures** | | | | | | | | |
| *Staphylococcus pseudintermedius (n=1)* | 0 | 1 | | 0 | n/a | n/a | n/a | n/a | |
| *Enterococcus spp. (n=2)* | 2 | 0 | | 0 | n/a | n/a | n/a | n/a | |
| *Escherichia coli (n=1)* | 1 | 0 | | 0 | n/a | n/a | n/a | n/a | |
